# SimplySmart_v1, a new tool for the analysis of DNA damage optimized in primary neuronal cultures

**DOI:** 10.1186/s12859-024-05947-8

**Published:** 2024-10-01

**Authors:** Sushma Koirala, Harman Sharma, Yee Lian Chew, Anna Konopka

**Affiliations:** Flinders Health and Medical Research Institute, College of Medicine and Public Health, Flinders University, Adelaide, Australia

**Keywords:** DNA damage foci, Neurodegeneration, DNA damage in neurons, Neuronal culture, Analysis, Python application

## Abstract

**Background:**

The increased interest in research on DNA damage in neurodegeneration has created a need for the development of tools dedicated to the analysis of DNA damage in neurons. Double-stranded breaks (DSBs) are among the most detrimental types of DNA damage and have become a subject of intensive research. DSBs result in DNA damage foci, which are detectable with the marker γH2AX. Manual counting of DNA damage foci is challenging and biased, and there is a lack of open-source programs optimized specifically in neurons. Thus, we developed a new, fully automated application, SimplySmart_v1, for DNA damage quantification and optimized its performance specifically in primary neurons cultured in vitro.

**Results:**

Compared with control neurons, SimplySmart_v1 accurately identifies the induction of DNA damage with etoposide in primary neurons. It also accurately quantifies DNA damage in the desired fraction of cells and processes a batch of images within a few seconds. SimplySmart_v1 was also capable of quantifying DNA damage effectively regardless of the cell type (neuron or NSC-34). The comparative analysis of SimplySmart_v1 with other open-source tools, such as Fiji, CellProfiler and a focinator, revealed that SimplySmart_v1 is the most ‘user-friendly’ and the quickest tool among others and provides highly accurate results free of variability between measurements. In the context of neurodegenerative research, SimplySmart_v1 revealed an increase in DNA damage in primary neurons expressing abnormal TAR DNA/RNA binding protein (TDP-43).

**Conclusions:**

These findings showed that SimplySmart_v1 is a new and effective tool for research on DNA damage and can successfully replace other available software.

## Introduction

Unlike mitotic dividing cells, neurons are postmitotic and do not undergo the cell cycle [1]. These neuronal characteristics have specific implications for DNA damage and repair processes in neurons. It is generally accepted that neurons differ from other cell types in terms of their sensitivity to DNA damage and mechanisms of DNA repair, and further studies on these processes are needed [1]. DNA damage has emerged as a novel and significant contributor to neurodegeneration [2–4]. Interestingly, DNA damage is common in various neurodegenerative disease, including amyotrophic lateral sclerosis and frontotemporal dementia and Alzheimer’s disease [2–4]. Thus, studies on the role of DNA damage processes in neuronal physiology and pathophysiology have become important research topics.

A number of open-source software programs for quantifying DNA damage foci have been developed (for a review, please see [5]). However, these programs lack a user-friendly interface, are semiautomated, and require a certain level of bioinformatics knowledge. In general, not all of these methods measure parameters other than the number of foci and do not allow for the use of specific markers [5]. Thus, SimplySmart_v1 is a good alternative for quantifying DNA damage by users without image processing or bioinformatics knowledge. The application is simplified to provide quick analysis of batches of images and simultaneously analyse foci from challenging images. In addition, SimplySmart_v1 was designed to target specific needs of neuroscience research. For example, the transfection of primary neurons is more difficult; thus, the transfection efficiency is weaker than that of immortalized cell lines. This often requires the identification of transfected cells for analysis within a mixture of cultured cells. Similarly, the preparation of fully pure neuronal cultures from brain tissue is often impossible, and other cell types of the nervous system are present. These features force manual neuron selection, making the analysis tedious and exposed to human error.

In addition, a relationship has been noted between nucleus size and the magnitude of DNA damage [6]. For example, DNA damage is significantly correlated with nuclear size but not with DNA content in senescent fibroblasts [6]. Thus, DNA damage foci analysis performed only within a defined nucleus size may be beneficial.

SimplySmart_v1 is a fully automated wxPython application and does not require knowledge of bioimage informatics. DNA damage in neurons can be quantified on the basis of marker expression or nucleus size. The application recognizes a single neuronal nucleus in a batch of images without the need for manual cell selection or image selection and returns the number of DNA damage foci per neuron as well as the size of the identified foci as an average value of the foci size per neuron. Furthermore, the application returns results in the form of convenient CSV files and output images.

The ability of SimplySmart_v1 to quantify DNA damage was validated in neurons with pharmacologically induced DNA damage with etoposide, which revealed that, compared with control neurons, SimplySmart_v1 accurately recognized the increase in DNA damage. In addition, we tested the performance of SimplySmart_v1 using NSC-34 cells and found that SimplySmart_v1 is capable of quantifying DNA damage in both cell types, primary neurons and NSC-34 cells. Mutation of TDP-43, a critical protein in amyotrophic lateral sclerosis and frontotemporal dementia [7], induces more DNA damage compared to wild-type TDP-43 neurons, when treated with etoposide. This finding is consistent with previous studies on the role of the TDP-43 protein in DNA damage in amyotrophic lateral sclerosis [8], confirming the validity of the described tool. While SimplySmart_v1 is a powerful and effective tool for the study of DNA damage in neuronal physiology and pathophysiology, it can also be successfully applied to other cell types.

## Implementation

### Step-by-step manual

**Fig. 1:**
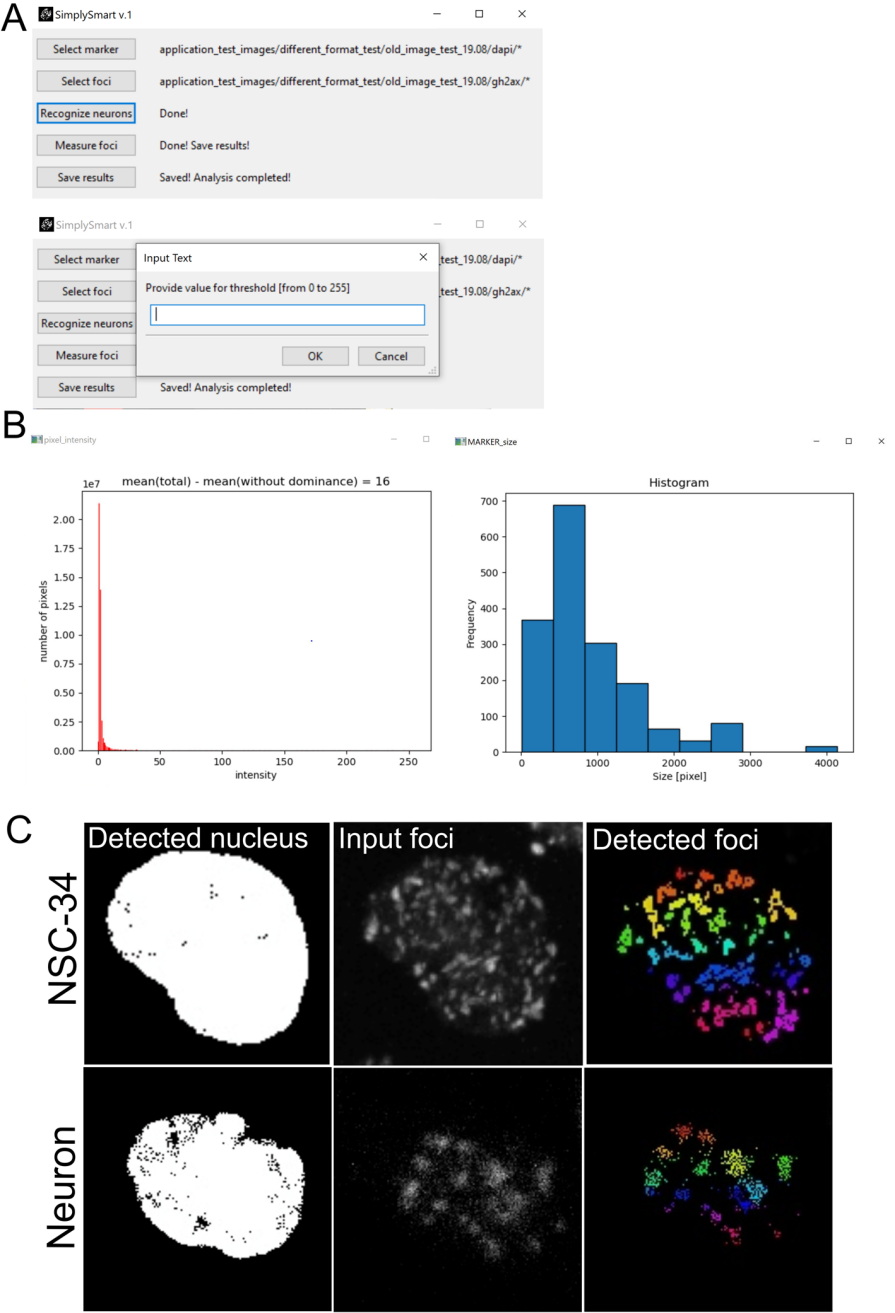
SimplySmart_v1 GUI interface. **A** SimplySmart_v1 has a user-friendly interface with 5 buttons appearing one by one once the previous task is completed. The user communicates with the application via the input text box. **B** Example of a ‘pixel_intensity’ graph (left) representing the pixel intensity distribution of the DNA damage channel for all analysed images. Example of a histogram ‘MARKER_size’ representing the size distribution of detected objects (right). **C** Examples of the detected DNA damage foci in motor neuron-like 34 (NSC-34) and mouse primary neurons

SimplySmart_v1 has a simple and user-friendly GUI, as shown in Fig. 1. The test data used in the project are available at https://github.com/AnnaEKonopka/SimplySmart_v1_materials.git.

To clone the repository, the following command is used:

git clone < URL to the repository > 

*Step 1* Prepare folders with images to be analysed. Images for each channel should be placed in separate folders. SimplySmart_v1 accepts the following image formats: JPEG, PNG, and TIFF.

*Step 2* The application is run, and the button on the interface is pressed to provide a path to folder with the MARKER images. These images are used for the recognition of neurons to be analysed (for example, DAPI- or NeuN-stained neurons). Copy and paste absolute path to the folder. Clicking OK, the next button will appear on the interface.

*If a new button does not appear, check if the provided path is correct. The new button will appear only if the data and path to the data are provided correctly*.

*Step 3* Click on the ‘Select foci’ button and provide a path to folder with the DNA damage images. Click OK, and the next button will appear.

*Similarly, if a new button does not appear, check if the provided path is correct. A new button will appear only if the path and data are provided correctly*.

With this action, two graphs appear: (1) a representation of the distribution of the pixel intensity, which is calculated as a sum of pixels about specific intensity for all the input images, and (2) a histogram representing the size of the detected objects (MARKER).

*Step 4* Click on the new button ‘Recognize neurons’. You will be asked to provide (i) a value for thresholding in the range from 0 to 255 (this can be determined on the basis of the displayed graph ‘pixel_intensity’, the graph flattening allows us to determine the cut-off point for background, in addition to that the suggested value for the minimal cut-off point is displayed as a difference between the mean number of pixels and the mean number of pixels calculated after subtraction of dominance), (ii) the minimum and maximum areas of MARKER to be considered in the analysis in pixels (this can be determined on the basis of the displayed histogram of ‘MARKER_size’), and (iii) the minimal area of foci to be analysed in pixels (user choice, type 1 if there is no specific requirements for foci size). Click OK. The application passes through all images to detect neurons/nuclei with specified parameters. Once completed, a new button will appear.

*If you do not see ‘Done!’ and a new button does not appear, check if your folders with images are correct, for example, if they contain an equal number of images for each channel and are in the correct format.*

*Step 5* Click on the new button ‘Measure foci’. Now, the application recognizes DNA damage foci filtered in Step 4 nuclei. The last button will appear.

*If you do not see ‘Done! Save results!’ and new button does not appear, check if your folders with images are correct, for example contain equal number of images for each channel and in correct format.*

*Step 6* Clicking on the button ‘Save results’, you will be asked to provide the name for your output folder. Use name, which will describe your set of data specifically, do not use name ‘Results’ as this name is used by the application. Now, the analysis is complete, and you will find your output folder within the application folder. Your output folder will contain three folders with output images: (i) ‘Output_images_nucleus’ (with detected nuclei/neurons), (ii) ‘Output_images_DNAdamage’ (with detected DNA damage), and (iii) Output_images_overlaid (with merged raw images and detected DNA damage foci). You will also obtain the CSV file ‘Given_parameters’ with parameters provided by you and the CSV file ‘Quantification’ with the results.

*The output images can be visually inspected to exclude potential clumped nuclei or nuclei located at the edge of the image. However, the well-defined range of MARKER sizes to be analysed allows us to avoid or reduce such occurrence.*

*Step 7* Close and run the application again to analyse another batch of images. Your output folder will stay intact in its original location.

## Methods

### Animal ethics and procedure

The timed pregnant mice were purchased from Flinders University Animal Facility. Prior to cervical dislocation, the mice were exposed to carbon dioxide. Specifically, 20% carbon dioxide was applied to induce anaesthesia, followed by an increase in the flow rate of carbon dioxide until respiration ceased. The mice were already unconscious after being supplied with carbon dioxide and did not suffer due to the procedure. After isolation, the embryos were immediately humanely killed via decapitation. This method enables one to reduce animal pain and distress to the minimum and, at the same time, to obtain high-quality primary neuronal cultures. All procedures were performed in accordance with *the Australian Code for the Care and Use of Animals for Scientific Purposes, 8th edn, 2013*, and were approved by the Flinders University Animal Ethics Committee.

### Primary neuronal cultures

Primary cortical neurons were isolated from C57BL/6 E16 mouse brains, plated on glass coverslips covered with poly-L-lysine and cultured in media supplemented with B27 (Gibco, cat # 17504044), neurobasal media (Gibco, cat # 10888022), 1% GlutaMAX (Gibco, cat # 35050061) and 1% penicillin/streptomycin (Gibco, cat # 15140122). The neurons were incubated at 37 °C and 5% CO_2_ until they were subjected to experiments at 3 or 5 DIV (days in vitro).

### NSC-34 cultures

NSC-34 cells were cultured in DMEM (Gibco, cat # 10569010) with 10% FBS (Gibco, cat # A3160401) at 37 °C and 5% CO_2._

### DNA damage induction

DNA damage was induced by treatment with 13.5 μM etoposide (Sigma Aldrich, cat # E1383-25MG) for 1 h. Dimethyl sulfoxide (DMSO) (Sigma Aldrich, cat # D4540-100ML) was used as a control treatment.

### TDP-43 expression in primary neurons

Primary mouse cortical neurons were transduced with lentiviruses carrying wild-type TDP-43 or TDP-43 mutant A315T. pLVX-Puro-TDP-43-A315T was a gift from Shawn Ferguson (Addgene plasmid # 133755; https://www.addgene.org/133755/; RRID: Addgene_133755). Similarly, pLVX-Puro-TDP-43-WT was a gift from Shawn Ferguson (Addgene plasmid # 133753; https://www.addgene.org/133753/; RRID: Addgene_133753). The viruses were prepared at the Gene Silencing and Expression Core Facility, Adelaide University, South Australia.

### Immunostaining

Neurons were fixed in 4% paraformaldehyde and washed with PBS three times. Then, the cells were permeabilized in 0.1% Triton X-100 in PBS for 5 min. Nonspecific binding was blocked in 5% BSA in PBS for 30 min. Staining was performed overnight with primary antibodies, including rabbit anti-γH2AX (Bethyl Laboratories, cat # A700-053), mouse anti-NeuN (Invitrogen, cat # MA533103) or mouse anti-TDP-43 (Invitrogen, cat # MA527828), at a concentration of 1:500. The samples were incubated with the following secondary antibodies for 2 h at room temperature: anti-mouse/rabbit Alexa Fluor 488 (Invitrogen, cat # A-11001, cat # A-11070) or anti-mouse/rabbit Alexa Fluor 594 (Invitrogen, cat # A-11005, cat # A-11012). The nuclei were counterstained with DAPI.

### Image acquisition

Fluorescently labelled cells were acquired via an Olympus AX70 microscope or Zeiss LSM 880 confocal microscope. The images were saved in JPEG or TIFF format and placed in separate folders for each single channel (γH2AX, DAPI, NeuN or TDP-43). No image preprocessing was performed before analysis.

### Thresholding and foci quantification

The background cut-off value was established for each experiment according to the data. The minimal pixel number describing the size of the DNA damage foci to be analysed was set at > = 1 to minimize potential false-positive recognition of noise. For the recognition of neurons, the application uses OTSU thresholding and connected component functions, which are available in the OpenCV package. For the identification of DNA damage foci within recognized neurons, the adaptive Gaussian C threshold and connected component function available within the OpenCV package were utilized.

### Statistics

The data were analysed with Student’s t or one-way ANOVA follow by Tukey’s post hoc test. The statistical analyses were performed with GraphPad Prism 7 software, and a *p* value < 0.05 was considered to indicate statistical significance.

## Results

### SimplySmart_v1 accurately recognizes DNA damage and processes a large batch of images

Treatment of cells with etoposide induces DNA damage, which can be detected by staining for γH2AX, a marker of DSBs. The signal for γH2AX forms characteristic foci at the site of DNA breaks. First, the application was tested on a small set of images, including 3 control images and 4 images of etoposide-treated mouse primary neurons. After induction of DNA damage with etoposide (ETOP), mouse cortical neurons were stained for γH2AX to detect DNA damage and stained with DAPI to visualize the nucleus. The analysis was performed using neurons with a nuclear area in the range of 1000–1300 pixels. Nuclei on the edges of images or clumped nuclei were excluded from the statistical analysis to improve accuracy and reduce potential errors. In total, 13 control DMSO-treated and 8 etoposide-treated neurons were included in the analysis.

**Fig. 2:**
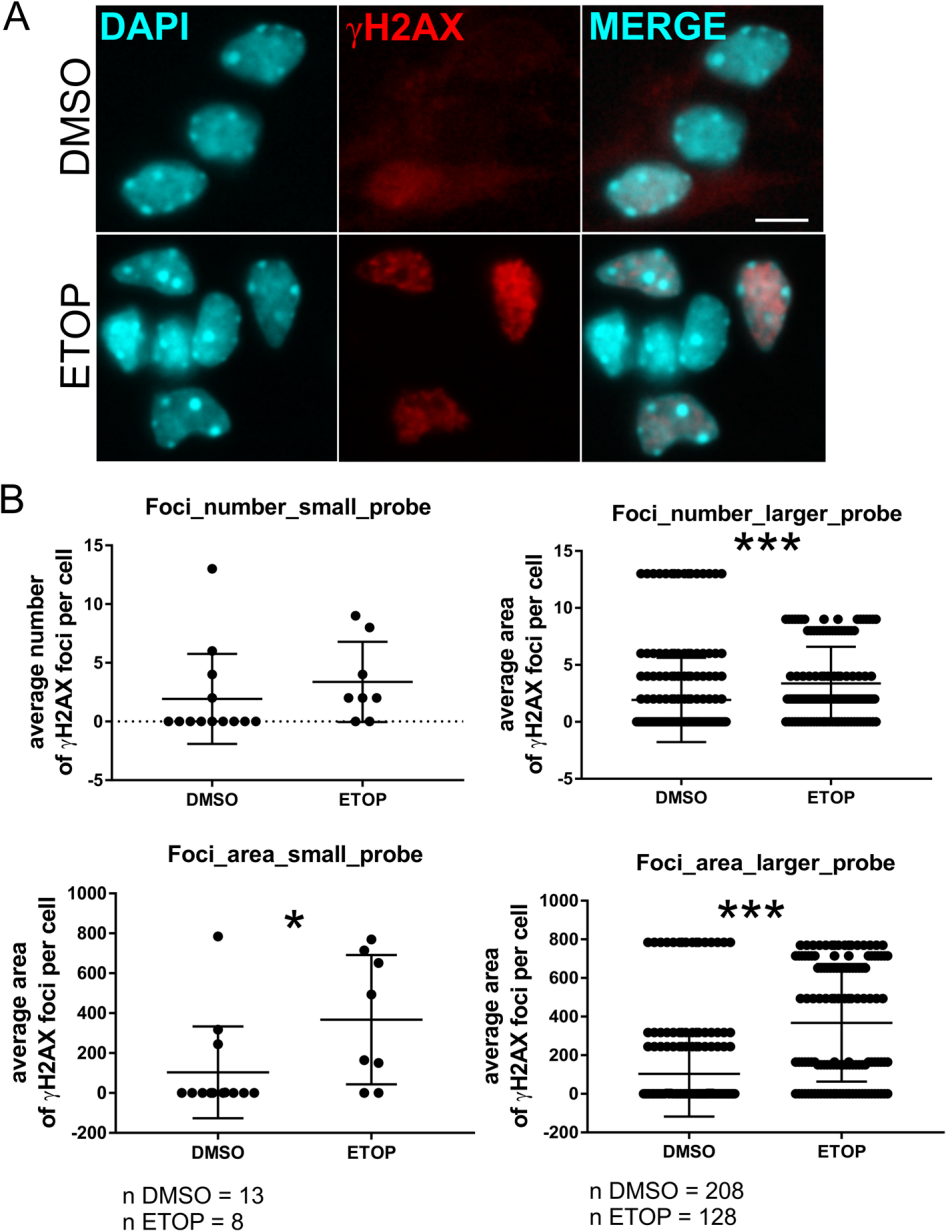
SimplySmart_v1 revealed the increase of DNA damage in mouse cortical neurons after etoposide treatment. **A** Representative images of mouse cortical neurons stained for the DNA damage marker γH2AX (red). Nuclei were visualized with DAPI (cyan). Upper row, DMSO-treated neurons; lower row, etoposide (ETOP)-treated neurons. **B** Graphs showing the average number and area of damaged DNA per neuron obtained from a small set of images (left graphs) and the average number and area of damaged DNA foci per neuron obtained from multiple copies of small set images (right graphs). *p < 0.05, ***p < 0.001, t test

DNA damage was greater in the ETOP group than in the DMSO group. Although this small probe did not significantly affect the number of foci (average number of foci: DMSO = 1.92, ETOP = 3.37, *p* > 0.05, t test, Fig. 2), the area of DNA damage foci was significantly greater in the ETOP group than in the DMSO group (average area of foci: DMSO = 103.6, ETOP = 367.6, *p < 0.05, t test, Fig. 2).

To test the application further, the measurements were performed on a large batch of images obtained by multiplication of the same images (16 × copies). This resulted in 48 single images for the DMSO group (for each channel) and 64 images for the ETOP group (for each channel). Despite the large amount of data available for processing, the application quantified DNA damage within a few seconds and generated data with the same quality as that obtained on the original small set. After visual inspection of the images, the incorrectly recognized single cells were removed as previously described. As expected, the results reflected an increase from the single small set with 208 cells analysed for the DMSO group, with 16-fold more results than the single set and the same average value = 1.92 foci per cell, and 128 cells analysed for the ETOP group, corresponding to 16-fold more results than the single set and the same average value = 3.37 foci per cell. Compared with that in the DMSO control group, the number of DNA damage foci in the ETOP group was significantly greater (***p < 0.001, t test, Fig. 2). Similarly, the area of DNA damage foci was significantly greater in the ETOP group (average area = 367.6) than in the DMSO control group (average area = 103.6) (***p < 0.001, t test, Fig. 2).

Thus, the application accurately detected an increase in DNA damage in ETOP-treated neurons compared with that in DMSO-treated neurons and was capable of analysing large batches of images.

### SimplySmart_v1 accurately recognizes DNA damage foci in NSC-34 cells

**Fig. 3:**
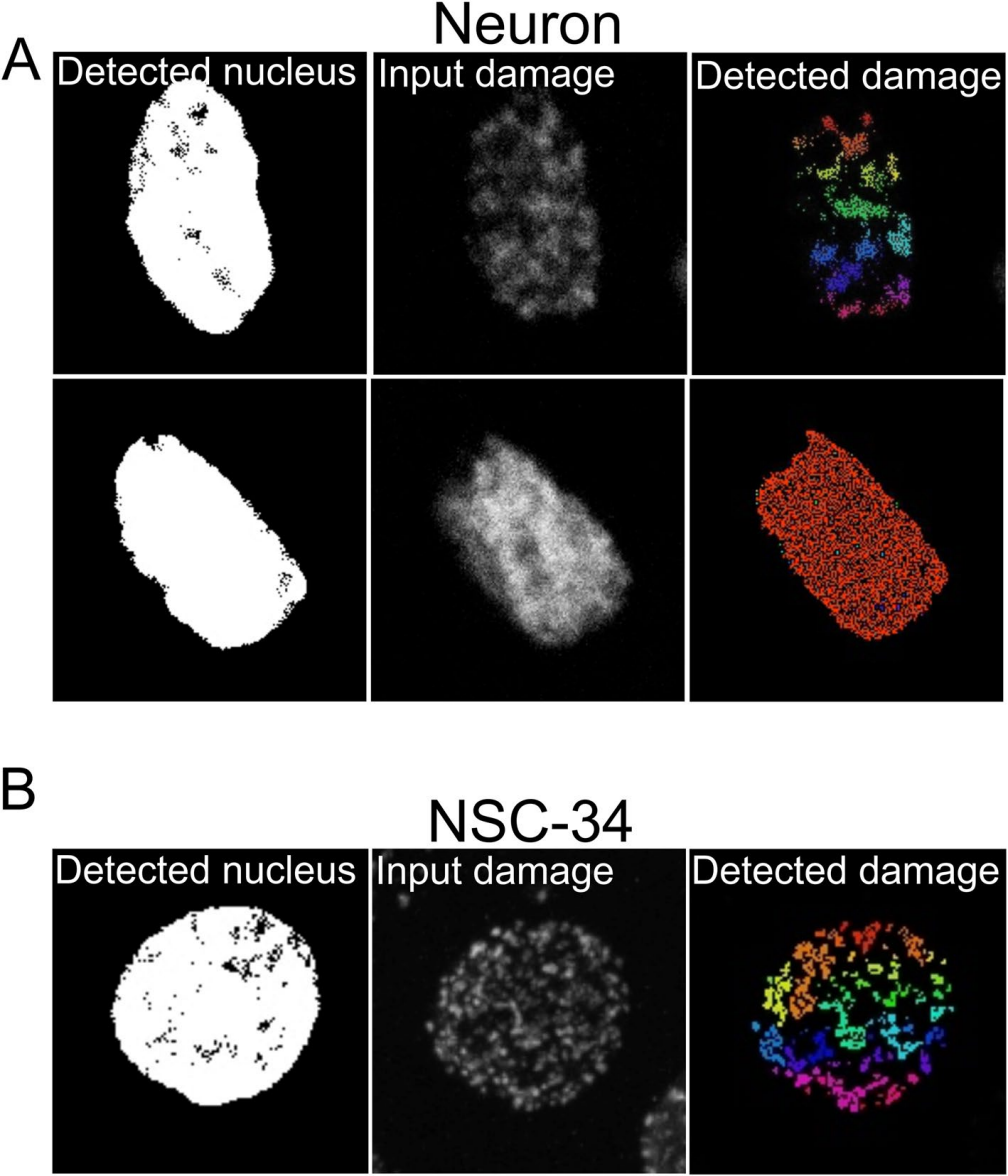
Representative images of DNA damage recognized in mouse primary neurons (**A**) and NSC-34 cells (**B**) by SimplySmart_v1. **A** Substantial heterogeneity in the appearance of DNA damage was observed across mouse primary neurons. **B** Representative image showing DNA damage foci in NSC-34 cells recognized by SimplySmart_v1

Next, we tested the performance of SimplySmart_v1 on an immortalized cell line, NSC-34, which was treated with etoposide. Interestingly, we observed less heterogeneity in the appearance of DNA damage foci in NSC-34 cells than in primary mouse neurons, even after treatment with the same dose of etoposide (13.5 µM). Examples of more structured foci and pannuclear damage in neurons are shown in Fig. 3A. In both cases, SimplySmart_v1 was capable of identifying damaged regions (Fig. 3A). However, we noted that, in contrast to NSC-34 cells, the analysis performed on primary neurons may require a few more adjustments of thresholding.

Similar to primary neurons, SimplySmart_v1 recognized DNA damage foci in NSC-34 cells with high accuracy (Fig. 3B). These findings imply that although SimplySmart_v1 was optimized in mouse primary neurons, it can be successfully used in the analysis of DNA damage in other cell types.

### SimplySmart_v1 quantifies DNA damage in the desired fraction of cells on the basis of the size of the nucleus

**Fig. 4:**
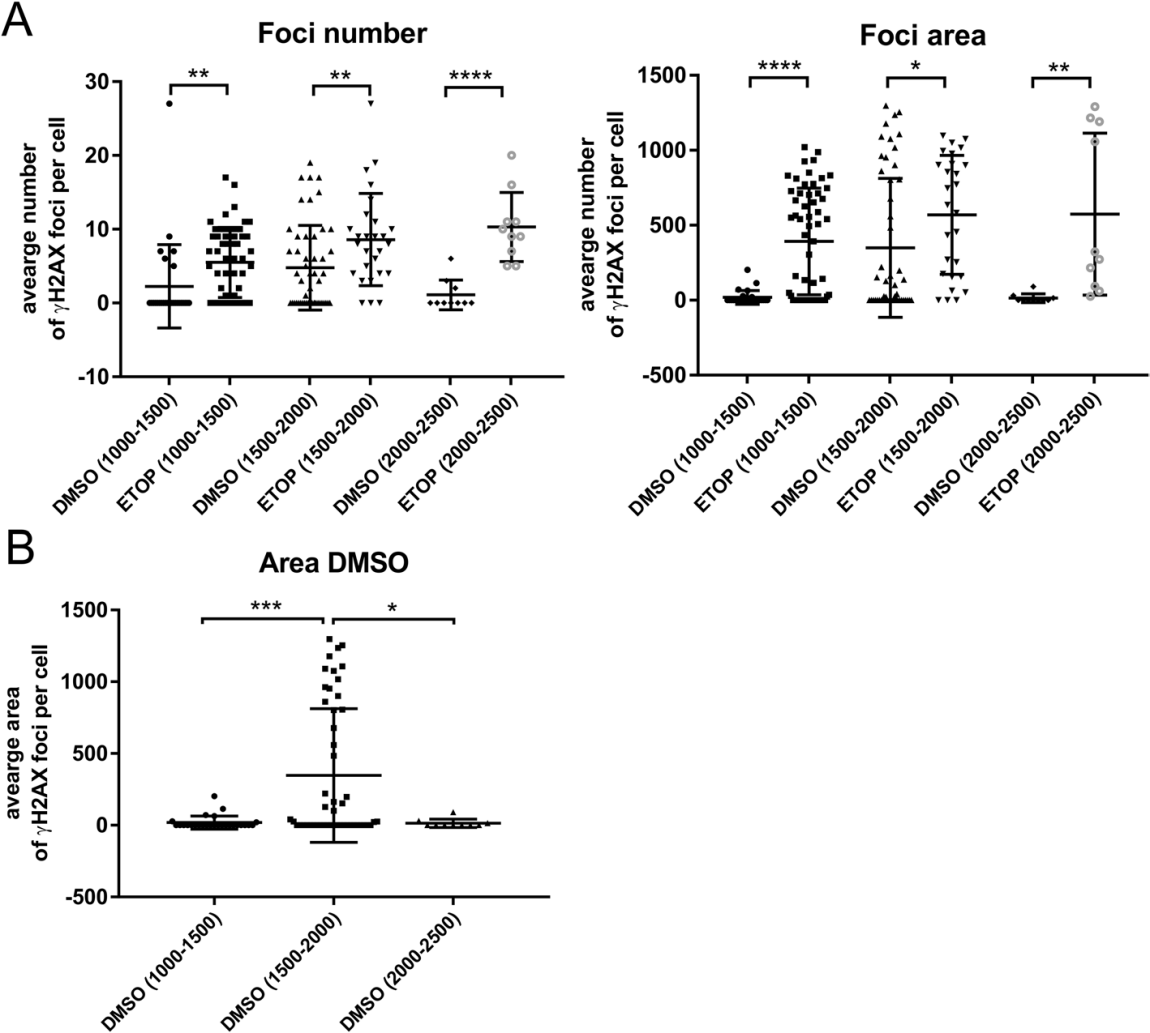
The nucleus size impacts the magnitude of DNA damage. **A** Quantification of foci number and foci area within different ranges of nucleus size revealed the variability in the magnitude of the increase in DNA damage after etoposide treatment compared with that under DMSO control conditions (foci number, **p < 0.01, **p < 0.01 or ****p < 0.0001 was reached depending on the nucleus size, 1000–1500, 1500–2000, 2000–2500 pixels, respectively; foci area, ****p < 0.0001, *p < 0.05 or **p < 0.01 was reached depending on the nucleus size, 1000–1500, 1500–2000, 2000–2500 pixels, respectively). **B** The size of the nucleus affected the magnitude of DNA damage in the DMSO-treated group. The area of DNA damage in cells within the nucleus size range of 1500–2000 pixels was significantly larger than that in the other groups, ***p < 0.001 vs. the 1000–1500 pixel group and *p < 0.05 vs. the 2000–2500 pixel group; one-way ANOVA with Tukey’s post hoc test was used

Among neurons, other cell types, such as astrocytes, oligodendrocytes or microglia, are often present in primary neuronal cultures. The staining of a specific type of cell helps to identify the desired cell type. However, such staining is not always possible due to experimental requirements or the limited number of dyes. Moreover, the size of the nucleus may impact the magnitude of DNA damage [6]. Therefore, the ability of SimplySmart_v1 to analyse DNA damage in neurons, which were selected on the basis exclusively of nucleus size, was assessed. First, we investigated whether the size of the nucleus impacts the magnitude of DNA damage. Thus, we divided the nucleus size into three groups, 1000–1500, 1500–2000, and 2000–2500 pixels, on the basis of the histogram generated by SimplySmart_v1. We then compared the number of foci and total area of foci measured in DMSO-treated or ETOP-treated cells with SimplySmart_v1 (Fig. 4). In all the groups, the ETOP-treated cells presented more DNA damage than did the DMSO-treated cells for both parameters, as expected. However, these differences were more prominent in the last group (2000–2500 pixels) than in the other groups for the number of foci (****p < 0.0001 vs. **p < 0.01 and **p < 0.01, t test) and in the first group (1000–1500 pixels) than in the other groups for the foci area (****p < 0.0001 vs. *p < 0.05 and **p < 0.01, t test). This implies that the nucleus size impacts the magnitude of the change between the control and experimental conditions (Fig. 4A). Interestingly, the nucleus size affected the magnitude of DNA damage in the DMSO control group itself, as the area of DNA damage in the DMSO-treated group (1500–2000 pixels) was significantly larger than that in the other DMSO-treated groups (1000–1500 and 2000–2500 pixels, ***p < 0.001 and *p < 0.05, respectively; one-way ANOVA, Tukey’s post hoc test) (Fig. 4B). Hence, the influence of nucleus size on the results may be particularly important in the case of less prominent changes in DNA damage, e.g., in experimental settings without pharmacological induction of DNA damage.

To further test the utility of the option of nucleus size selection provided by SimplySmart_v1, we determined whether it is possible to filter neurons on the basis of only the nucleus size. The size of the neuronal nucleus was determined via 8-bit images displaying DAPI staining and the neuronal marker NeuN with FijiJ software. The values are expressed in pixels. The quantification of 11 nuclei from two random images revealed that the area of the neuronal nucleus ranged between 1273 and 2534 pixels. Thus, this range was used in the analysis using SimplySmart_v1. Only single and whole nuclei were included in the statistical analysis. The application recognized 74 nuclei within the DMSO group and 69 nuclei within the ETOP group; one nucleus was excluded from further analysis because it was a clump of two nuclei.

The accuracy of neuron selection on the basis of nucleus size was subsequently verified with manual counting. Thus, the size of the nuclei in the images was measured with respect to the expression of the neuronal marker NeuN using Fiji J software. In the control DMSO group, 75 cells with clear expression of the marker NeuN and a nucleus size ranging from 1167 to 2504 pixels were obtained. Only 3 neurons were outside of the previously identified size range between 1273 and 2534, accounting for 4% of the NeuN-stained cells.

In addition, 12 cells with weak NeuN expression were obtained within both sizes ranging from 1167 to 2504 and from 1273 to 2534, and 5 cells with a lack of NeuN expression and a nucleus size below these ranges were observed. Moreover, 4 cells with a lack of NeuN expression and a nucleus size above these size ranges were noted, and one likely apoptotic cell with remnants of NeuN expression and a nucleus size below this size range was identified.

**Fig. 5:**
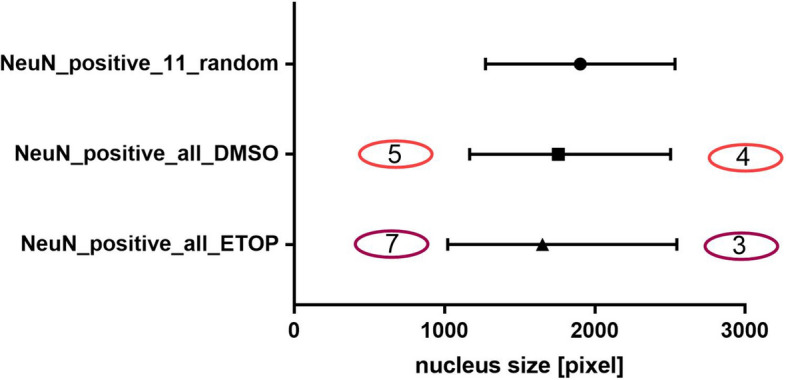
The nucleus size of the NeuN-positive cells was within an initially defined range, but that of the NeuN-negative cells was outside this range. The graph shows the ranges of nucleus sizes of NeuN-positive cells determined by counting 11 random cells with NeuN expression vs. determining the nucleus size of all cells with respect to NeuN expression. Overall, these calculations revealed that the number of NeuN-positive cells fell within the initially defined nucleus size range, whereas the nucleus size of NeuN-negative cells was outside of this range. The numbers in oval shapes indicate the number of NeuN-negative cells with nucleus sizes outside the range

In etoposide-treated neurons, 83 cells with NeuN expression and nuclear sizes ranging between 1020 and 2546 pixels were observed. Of these, 10 neurons were outside of the previously defined size range of 1273–2534 pixels, for a total of 12.1%. Five nuclei within the size range with weak expression of NeuN, 7 nuclei below the range without NeuN expression, and 2 nuclei above the range without NeuN expression were observed (Fig. 5).

Thus, initial determination of nucleus size allowed the selection of NeuN-positive cells with high precision and the simultaneous removal of cells with a clear lack of NeuN expression using SimplySmart_v1.

### The results obtained with SimplySmart_v1 and manual counting are consistent

**Fig. 6:**
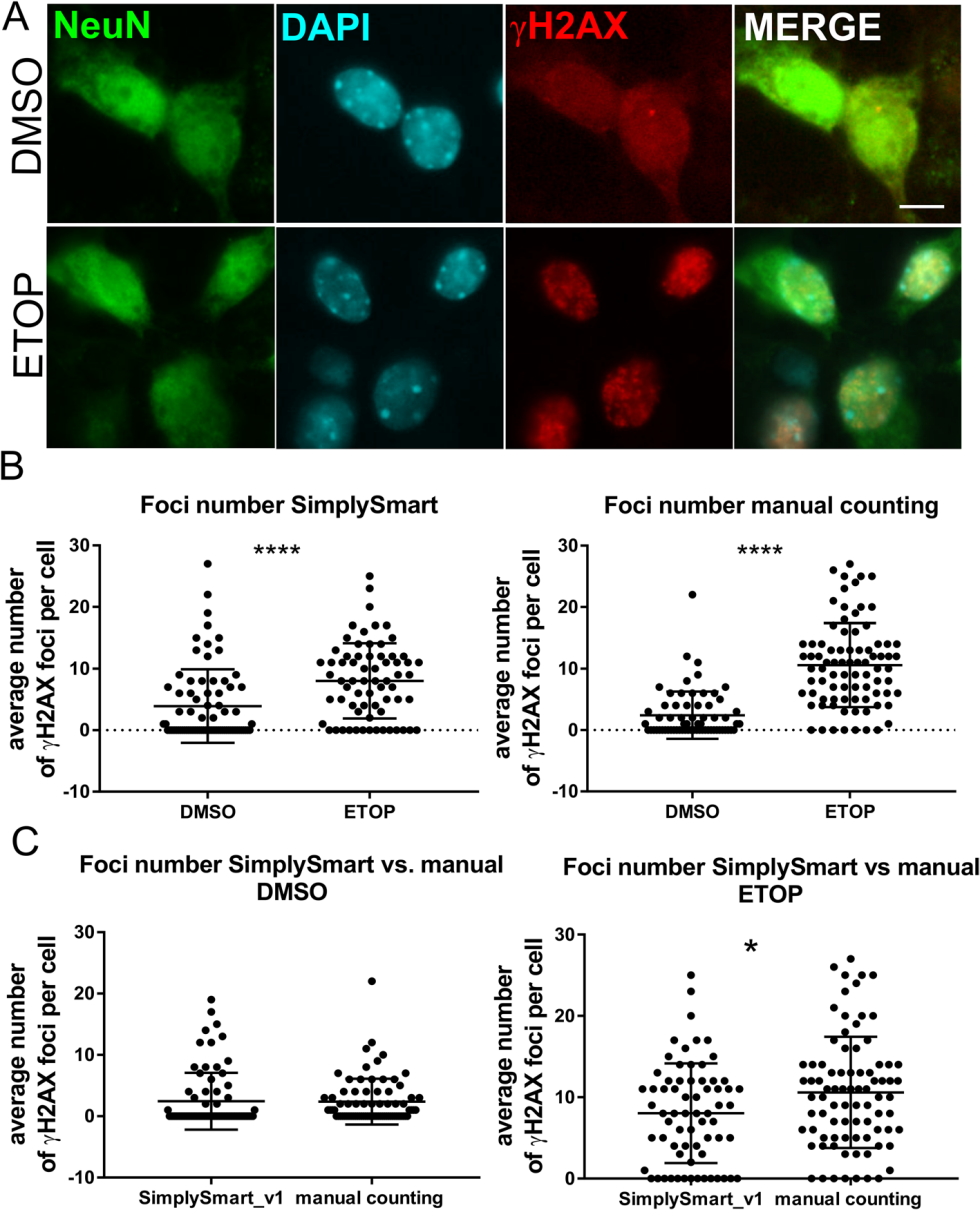
Manual validation of the data revealed the accuracy of the data obtained with SimplySmart_v1. **A** Representative images of mouse cortical neurons stained for the neuronal marker NeuN (green), the DNA damage marker γH2AX (red) and nuclei (cyan). **B** Graphs showing data obtained with the SimplySmart_v1 application (left) and manual counting (right). Compared with the DMSO control group, the ETOP group presented an increase in the average number of DNA damage foci (****p < 0.0001, t test). **C** Comparison of the data obtained with SimplySmart_v1 and manual counting. Left panel, DMSO group (*p* > 0.05); right panel, ETOP group (*p < 0.05, t test)

Next, the accuracy of the DNA damage measurements performed with SimplySmart_v1 was compared with that of manual counting for the above data. SimplySmart_v1 revealed that the average number of foci per neuron was 3.92 for the control group and 8.02 for the etoposide group, with a statistically significant difference of ****p < 0.0001 according to the t test. The average total area of DNA damage per cell was 229.1 for the control group and 467.6 for the etoposide group, with a statistically significant difference of ***p < 0.001 according to the t test (Fig. 6 A and B, the foci area is not shown as it cannot be compared with manual counting results).

The number of DNA damage foci for NeuN-positive cells obtained by manual counting was 2.45 foci/cell in DMSO-treated neurons with clear NeuN expression and 10.59 in ETOP-treated neurons, with a statistically significant difference of ****p < 0.0001 according to the t test (Fig. 6A and B). Thus, manual validation of the data confirmed that SimplySmart_v1 accurately assessed increased DNA damage in neurons treated with etoposide.

In addition, no differences were noted in the values obtained for the DMSO groups using manual counting and SimplySmart_v1 (*p* > 0.05, t test). However, the ETOP groups differed with *p < 0.05 (*p* = 0.03; t test) (Fig. 6C). Because visual DNA damage foci counting is more vulnerable to bias and human error, it is difficult to determine the actual reason for this discrepancy. Nevertheless, both manual counting and SimplySmart_v1 revealed an increase in the number of DNA damage foci after etoposide treatment, which was consistent with our findings.

### Comparison of SimplySmart_v1 with Fiji, CellProfiler and the focinator

**Fig. 7:**
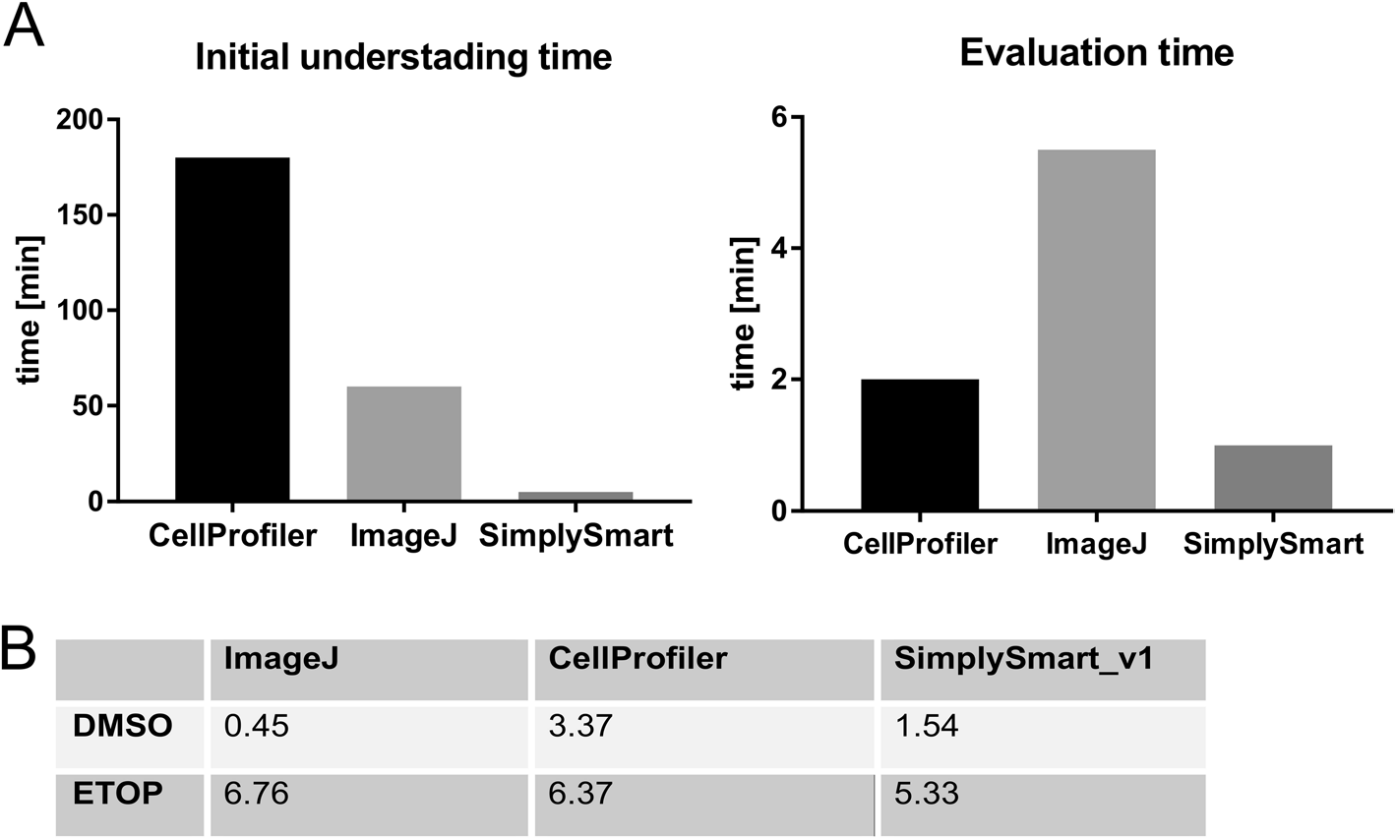
The open-source software comparison advantages SimplySmart_v1 over other tools. **A** Compared with Fiji and CellProfiler, SimplySmart_v1 requires the shortest time to comprehend the usage of the software (left) and to process the images (right). **B** Average number of γH2AX foci per nucleus obtained with different tools on the same set of images

We tested the utility of SimplySmart_v1 in comparison with other open-source tools, such as Fiji (Fiji-win32) [9], CellProfiler (4.2.5) [10] and a focinator [11]. The tests were performed by a person without background in image analysis to determine the simplicity of these tools. In total, 6 images of DMSO-treated neurons and 6 images of etoposide-treated neurons were analysed. The evaluation was performed on the basis of the number of γH2AX foci. The use of CellProfiler needed a significant amount of time for initial learning and understanding of the software. The configuration and development of the pipeline was a complex process, and an accurate understanding of the software was needed. This software was found to be a better option for experienced programmers than for novice users. Compared with CellProfiler, Fiji required less time for initial understanding but required more time than did SimplySmart_v1 (Fig. 7A).

Analysis with Fiji required at least 4.5 min to analyse the DMSO group and 6.5 min to analyse the ETOP group. Similarly, approximately 2 min per group was required for CellProfiler as a first-time user of the software. The analysis performed with SimplySmart_v1 was exceptionally rapid and took approximately 1 min without any specific training prior to the analysis. Although all tools performed the analysis relatively quickly, evaluating the time spent on training as well as the actual analysis advantages SimplySmart_v1 (Fig. 7A).

We also undertook an attempt to test the focinator, however, the installation turned out to be too challenging. Notably, this requires a specific version of Fiji (Fiji 1.51 bundled with Java, the Bio-Format importer, and the R-3.5.1 language), a basic understanding of the bioformats package Jar and R language. An individual without a related background found it too difficult and complex. Therefore, we focused on comparing SimplySmart_v1 with Fiji and CellProfiler.

Both Fiji and CellProfiler performed well; however, the accuracy of the foci count with Fiji might be affected when the images have weak contrast between the background and objects of interest. Fiji enabled the acquisition of information such as total area, average size of the foci, % area and mean value.

The effectiveness of CellProfiler is heavily dependent on pipeline creation. In accordance with the instructions in the CellProfiler manual, an example pipeline for speckle counts was used. However, the configuration problem of the inability of the pipeline to identify image sets was encountered several times as a first-time user of the software. Additionally, building a pipeline from scratch was a challenging procedure. Despite having several challenges, more information, such as the execution time to identify the targeted objects or object intensity, is provided by CellProfiler.

In contrast, the analysis with SimplySmart_v1 was straightforward and effective and was supported by a very user-friendly interface. While Fiji is also user-friendly, the procedure is arduous and time-consuming. The ‘user-friendly’ aspect was found to be the greatest drawback of CellProfiler and focinator.

All tools were capable of detecting increased amounts of DNA damage in the tested ETOP group compared with the DMSO control group. Figure 7B shows the analysis of the average number of foci per nucleus quantified with all three software programs. The analysis revealed that the average quantification result deviated between the DMSO control and ETOP treated groups, with the variation being lower in the ETOP treated groups among all three tools. Because of the variation, highly consistent results were obtained with SimplySmart, whereas the susceptibility to changes in quantification was observed with respect to Fiji and CellProfiler. This confirms the reliability of the quantification performed with SimplySmart_v1.

### Utilization of the full capabilities of SimplySmart_v1 showed induction of DNA damage in neurons expressing mutant TDP-43 (A315T) compared to wild type TDP-43

**Fig. 8:**
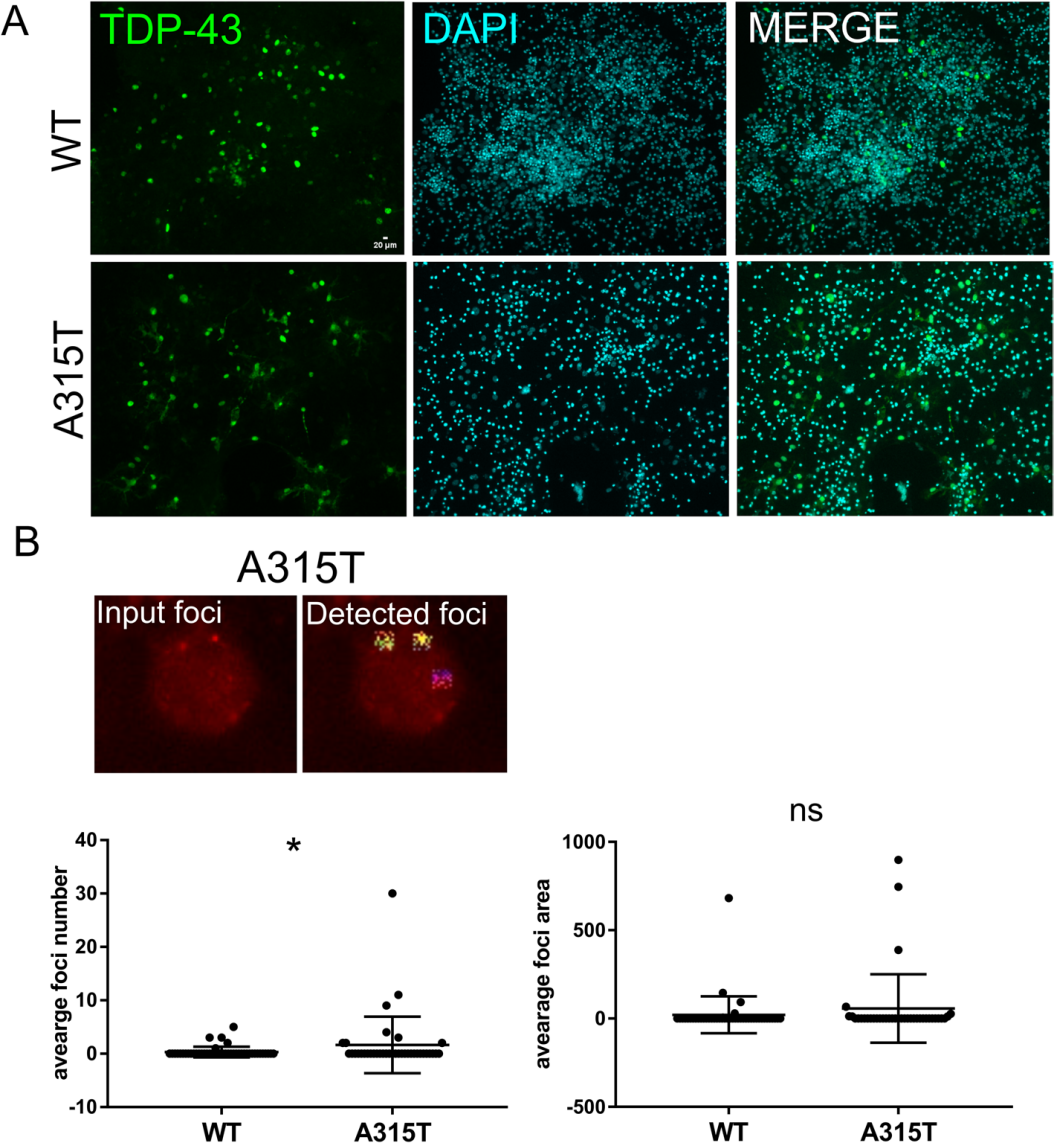
Compared with WT TDP-43, TDP-43 mutant A315T induced more DNA damage, as calculated with a one-sided t test. **A** Representative images of mouse cortical neurons stained with TDP-43 and DAPI. The selection of cells treated with puromycin resulted in many apoptotic nuclei; however, staining for TDP-43 revealed surviving transduced neurons. **B** The upper panel shows DNA damage areas identified using SimplySmart_v1 in neuronal nuclei expressing A315T (color-mapped regions). The graphs show that the average number of DNA damage foci in the WT TDP-43 group was significantly lower than that in the A315T group (*p < 0.05, one-sided t test), whereas the foci area was unchanged. The images were analysed with SimplySmart_v1 with a threshold value = 140, a marker area range of 1000–5000 pixels, and a minimal size > 2 pixels. Forty-nine nuclei from the WT group and 38 from the A315T group were analysed from 3 technical replicates

To further test the ability of SimplySmart_v1, the induction of DNA damage was quantified in mouse primary neurons expressing wild-type TDP-43 (WT) or its amyotrophic lateral sclerosis-associated mutant, A315T. Primary neurons were transduced with wild-type or A315T TDP-43 lentiviruses with puromycin as a selective marker. Treatment of neurons with puromycin resulted in a number of apoptotic cells, making the identification of a single nucleus on the basis of DAPI challenging. However, staining for TDP-43 allowed us to identify surviving, transduced neurons, and this staining was used as a marker in the analysis with SimplySmart_v1 (Fig. 8 A). DNA damage was detected by immunostaining for γH2AX as previously described. Only DNA damage foci larger than 2 pixels were counted. After visual inspection of the output images, clumps of cells or cells located at the edges of the images were excluded from the statistical analysis.

In our previous study, we reported that neurons expressing mutant TDP-43 A315T are characterized by increased DNA damage compared with those expressing wild-type TDP-43 after pharmacological induction of DNA damage with etoposide [8]. Here, we used SimplySmart_v1 to investigate whether neurons expressing TDP-43 A315T exhibit increased DNA damage compared with those expressing wild-type TDP-43 without any exogenous induction of DNA damage. Consistent with our previous findings, we did not observe a statistically significant difference in the magnitude of DNA damage between TDP-43 A315T neurons and wild-type TDP-43 neurons according to two-sided t test (*p* > 0.05). However, neurons expressing mutant TDP-43 A315T were characterized by a significantly greater number of DNA damage foci than were those expressing wild-type TDP-43 when a one-sided t test was applied (*p < 0.05, t test). No statistically significant differences in the area of DNA damage foci were noted between the investigated groups, regardless of the type of test used (two- or one-sided) (*p* > 0.05, t test) (Fig. 8 B). Although the data were obtained with different quantification techniques and in different experimental settings, they are consistent with our previous findings [8]. They also showed that the flexibility of SimplySmart_v1 makes it a powerful tool that is applicable for DNA damage analysis in real experimental settings.

## Discussion

Analysis of DNA damage is a time-consuming and challenging process. The automation of this process is an invaluable advancement that allows for timely, effective and unbiased results. Most of the available tools are complex, semiautomated or require bioimaging knowledge, making their use challenging for novice users. Given the increased interest in DNA damage research in neurodegeneration, we have developed a user-friendly and simple application, SimplySmart_v1, making it accessible to researchers without a background in bioimaging.

The experiments performed on primary neurons revealed that SimplySmart_v1 accurately quantified the magnitude of DNA damage in primary neurons after the induction of DNA damage with etoposide and under control conditions. The obtained results were validated by manual counting. However, in contrast to manual counting, SimplySmart_v1 has many more advantages because (i) it is quick and delivers results within a few seconds; (ii) is unbiased; and (iii) quantifies the area of DNA damage foci alongside the number of foci, a task not achievable with manual counting. Furthermore, this technique allows the quantification of DNA damage foci of a specific size (above the number of specified pixels). It also addresses the borders of DNA damage foci, which are difficult to recognize via the human eye.

A few studies suggest that the nucleus size impacts the magnitude of DNA damage [6, 12]. Our findings are consistent with these findings, showing variability between the control and etoposide-treated groups when different nucleus sizes were investigated. Primary neuronal cultures are typically prepared to obtain specific types of neurons, such as cortical or hippocampal neurons. While this approach aims for a uniform cell population, achieving a pure neuronal population can be challenging. SimplySmart_v1 supports neuron selection for analysis on the basis of nuclear size. We showed that SimplySmart_v1 can filter specific populations of cells on the basis of the nucleus size, providing more accurate results.

The methods used for experiments on primary neuronal cultures differ from those used for experiments on immortalized cell lines. For example, primary neurons are more difficult to transfect and are characterized by a lower transfection efficiency, requiring manual cell selection. SimplySmart_v1 overcomes this limitation and quantifies DNA damage foci in a fully automated manner. In addition, our study revealed that the induction of DNA damage in primary neurons is more heterogeneous than that in NSC-34 cells. Despite this, SimplySmart_v1 is capable of quantifying DNA damage in both cell types.

Finally, the increased interest in research on DNA damage in neurodegeneration has created the need for effective and simple tools for quantifying DNA damage. SimplySmart_v1 was assessed in actual research on neurodegeneration to investigate DNA damage in mouse primary neurons expressing wild-type TDP-43 or TDP-43 mutant A315T. TDP-43 is a critical protein involved in the pathogenesis of amyotrophic lateral sclerosis but also contributes to Alzheimer’s disease and frontotemporal dementia [7]. Using SimplySmart_v1 we showed increased DNA damage in neurons expressing TDP-43 mutant A315T compared to wild-type TDP-43 under baseline conditions without exogenous induction of DNA damage. These findings are consistent with our previous research [8]. Thus, SimplySmart_v1 meets the demands of DNA damage research in the context of neurodegeneration.

## Conclusions

This work describes the SimplySmart_v1 application and shows that SimplySmart_v1 is an effective tool for the quantification of DNA damage foci in neurons cultured in vitro. However, it can also be successfully applied to immortalized cell lines. The increased research interest in DNA damage in neuronal physiology and pathology, the simplicity of SimplySmart_v1 and complete automation of DNA damage analysis in neurons places SimplySmart_v1 to become a widely used tool in a broad research context.

### Availability and requirements

Project name: SimplySmart_v1 project; Project home page: https://github.com/AnnaEKonopka/SimplySmart_v1_materials.git; Operating system: Windows; Programming languages: python; Other requirements: None; Licence: wxWindows Licence, Open Source; Any restrictions to use by nonacademics: None.

## Data Availability

The data underlying this article are available in the article and in the online supplementary materials, GitHub repository, at https://github.com/AnnaEKonopka/SimplySmart_v1_materials.git.

## Abbreviations

TDP-43: TAR DNA/RNA binding protein
DMSO: Dimethyl sulfoxide
BSA: Bovine serum albumin
PBS: Phosphate-buffered saline
DAPI: 4′,6-Diamidino-2-phenylindole
ETOP: Etoposide
GUI: Graphical user interface
CSV: Comma separated values
DSBs: Double-stranded breaks
